# Efficient Separation of Oil/Water by a Biodegradable and Superhydrophobic Composite Based on Loofah and Rice Straw

**DOI:** 10.3390/membranes14110243

**Published:** 2024-11-18

**Authors:** Mamadou Souare, Changqing Dong, Tong Xing, Junjiao Zhang, Xiaoying Hu

**Affiliations:** 1 National Engineering Laboratory for Biomass Power Generation Equipment, School of New Energy, North China Electric Power University, Beijing 102206, China; generalsouare1@gmail.com (M.S.); xingkongvip@126.com (T.X.); xiaoying_826@163.com (X.H.); 2 State Key Laboratory of Alternate Electrical Power System with Renewable Energy Sources, North China Electric Power University, Beijing 102206, China; 3 Datang Environment Industry Group Co., Ltd., Beijing 100080, China; 4 School of Energy, Power and Mechanical Engineering, North China Electric Power University, Beijing 102206, China; zjunjiao@ncepu.edu.cn

**Keywords:** loofah, rice straw, oil/water separation, superhydrophobic

## Abstract

Membrane filtration is one of the preferred choices for petroleum wastewater disposal due to its simplicity and low energy consumption. In this paper, a biodegradable superhydrophobic membrane based on loofah and rice straw (LF-RS) was prepared and modified with dodecyltriethoxysilane to improve its stability, morphology, and performance. The membrane showed an efficiency of 99.06% for oil/water separation with an average water flux of 2057.37 Lm^−2^h^−1^ and a tensile strength of 11.19 MPa. The tensile strength of the LF-RS membrane was 322.47% higher than that of the PVDF membrane and 126.58% higher than that of the commercially available nitrocellulose membrane. Through molecular simulations, we showed a 96.3% reduction in interaction energy between water and membrane post-modification, which is beneficial for increasing the contact angle and separation performance. This study provides an option for the large-scale, cost-effective fabrication of eco-friendly membranes for pollutant removal.

## 1. Introduction

The leakage of crude oil and petroleum products might cause serious environmental pollution [1,2,3] and threaten human health [4,5]. Chemical treatments [6], burning, flotation, gravity separation [7,8], bioremediation [9], electrochemical processes [10], and membranes [11,12,13,14] have been used to treat oily wastewater. Among these, membrane filtration, especially for biomass-derived membranes, has become increasingly popular due to its efficiency, low energy consumption, and environmentally friendliness [15,16,17]. Bamboo residues [18], loofah [19,20], alginate [21], and fibers [22] have been used to construct superhydrophobic surfaces which minimizing fouling and increasing separation efficiency compared to superhydrophilic materials [8,9,10,11,12,13,14]. However, these biomass-derived membranes often encounter low mechanical robustness, environmental pollution, and high costs [23,24,25]. Additionally, exposure to acidic, alkaline, saline, or hot water might degrade the superhydrophobic properties and reduce the reusability of the material.

To overcome these limitations and improve the properties of the membrane, various additives, including fluorinated polybenzoxazine [26], nickel nanoclusters [27], polyvinylidene fluoride (PVDF) [28], silicon dioxide (SiO_2_) [29], Cu(OH)_2_ [30], titanium-dioxide (TiO_2_) [31,32], stearic acid [33], alkyl-ketenedimer [34], perfluorooctanoic acid, polydimethylsiloxane, perfluorooctyl triethoxysilane, and other fluorine-based compounds [35,36,37], have been used. However, these are either toxic or cause environmental problems when the membrane reaches its service life. In response, the use of natural and biodegradable materials for superhydrophobic membranes is drawing the attention of researchers [16].

In this paper, loofah and rice straw were selected as primary materials for membrane fabrication due to their sustainable properties. Loofah, with its unique porous structure, provides an ideal surface to retain oil while repelling water, thus enhancing oil/water separation efficiency. Rice straw, an abundant agricultural byproduct, adds mechanical stability to the membrane. Unlike previous studies [35,36,37] that relied on synthetic materials or fluorinated compounds, our approach leveraged natural materials to create a high-performance, eco-friendly membrane. A loofah- and straw-based membrane modified with dodecyltriethoxysilane was developed. The chemical stability of this membranes was thoroughly investigated and characterized, focusing on its resistance to challenging conditions, including exposure to acids, bases, various organic solvents (e.g., hexane, diethyl ether, dimethyl sulfoxide), and salts (e.g., potassium chloride, calcium chloride). The tensile strength and the effect of temperature were also evaluated. Furthermore, the interaction energies between water, oil, and the membrane were modeled using the molecular dynamics method to assess the impact of surface modifications.

## 2. Materials and Methods

### 2.1. Chemicals and Materials

Chemicals used for the membrane production, including dichloromethane, sodium hypochlorite, tetraethylorthosilicate, dodecyltriethoxysilane, and ethanol, were purchased from Shanghai Macklin Biochemical Co., Ltd., Shanghai, China. The rice straw/loofah composite was purchased from Yuan quan e-commerce Co., Ltd., Guangzhou, China. Olive oil, soybean oil, and kerosene were procured from a local store: Sanqibaihui shopping center, located in Beijing, China. The membranes of nylon (Amershan), nitrocellulose (NC, PALL), and polyvinylidene difluoride (PVDF, Millipore) used for comparative analysis were provided by Hengyuan Botai Biotechnology Co., Ltd., Xinyang, China.

### 2.2. Preparation of Superhydrophobic LF-RS Composite Membrane

**Figure 1:**
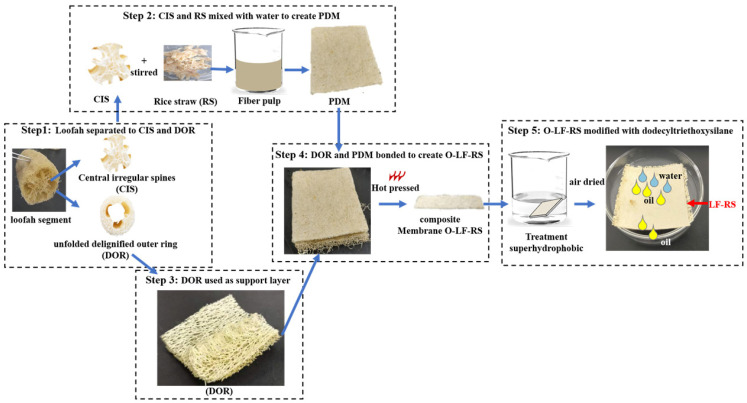
The fabrication process of the LF-RS membrane for water and oil separation.

The peeled loofah samples (10 cm in length, 6 cm in diameter) were boiled in water for one hour. The rice straw was cut into 3 cm long sticks. The boiled parts of the loofah (after air-drying) and rice straw sticks were soaked in a 5 wt% sodium hypochlorite solution at room temperature (23 °C) for six hours, followed by air-drying at 25 °C for 24 h. As shown in Figure 1, the treated loofah was separated into two parts: the delignified central irregular spine (CIS) and the unfolded delignified outer ring (DOR). The CIS of the loofah and processed rice straw (RS) were ground and mixed at a 1:1 (*w*/*w*) ratio, along with 250 mL of water. This mixture was poured into a container measuring 121.27 mm long, 81.52 mm wide, and 12 mm in height, then dried at 50 °C for 12 h to obtain the delignified pulp membrane (PDM). The PDM was superimposed on the DOR by hot pressing at 70 °C and 20 MPa. Finally, the original LF-RS membrane (O-LF-RS) was obtained. The O-LF-RS membranes were immersed in a dodecyltriethoxysilane and ethanol solution (dodecyltriethoxysilane: ethanol = 7.6: 270 mL; *v*/*v*) with 4.5 mL of orthosilicate-tetraethyl and stirred for thirty minutes. Subsequently, 5 mL of deionized water and 20 mL of ammonia were added to promote the condensation reaction of the silanol groups. After 24 h of soaking, the samples were cleaned with deionized water and ethanol, then air-dried at 50 °C for two hours to obtain the stable superhydrophobic LF-RS membrane.

### 2.3. Membrane Structure and Characterization

The structural and functional characterization of the membranes (O-LF-RS and LF-RS) was performed using Hitachi SU4800 (Hitachi High-Tech Corporation, Tokyo, Japan) scanning electron microscopy (SEM) and energy-dispersive spectrometry (EDS) (HORIBA Ltd., Kyoto, Japan). The functional groups of the membranes were analyzed with Fourier Transform Infrared (FT-IR) spectroscopy (Thermo Fisher Nicolet 6700, from Thermo Fisher Scientific, Waltham, MA, USA) in the range of 400–2000 cm^−1^. The membrane pore structure was evaluated using MicroActive AutoPore V 9620 (Norcross, GA, USA) (version 2.03.00, USA). Tear strength was assessed with an electronic tensile testing machine (Ningbo Weiheng Testing Instruments Co., Ltd., WH-5000, Ningbo, China) featuring a load transducer of 5 kN/100 N, using samples measuring 29 mm in length, 7 mm in width, and 0.89 mm in thickness.

### 2.4. Water Contact Angle (WCA) Test

The WCA on the membranes was measured with a contact angle goniometer (model JC2000D, Shanghai-Zhongchen Power Technology Co., Ltd, Shanghai, China.). A droplet of water (5 μL) was deposited on each LF-RS membrane, and the contact angle was determined by calculating the average of five measurements.

### 2.5. Thermogravimetric Analysis

The thermal stability of the LF-RS composite membrane samples before and after modification was analyzed using STA-FTIR-GC/MS (Curry model STA6000-Spectrum100-Clarus560, PerkinElmer, Inc., based in Waltham, MA, USA). Samples were heated from 25 to 550 °C at a heating rate of 5 °C/min. The calorimetric pressure was maintained at ±2%, with a mass variation sensitivity accuracy of 0.1 µg.

### 2.6. Surface Chemical Stability and Resistance

Drops of hydrochloric acid solutions (pH: 3, 5), water (pH: 7), and sodium hydroxide solutions (pH: 9, 11) were deposited on the surface of LF-RS samples for six hours with a dropped volume of 10 µL per sample to observe their wetting behavior [38,39,40,41,42]. The experiments were conducted under a controlled environment (temperature and humidity) to minimize these evaporation effects. To further evaluate the chemical stability of LF-RS samples, they were submerged in corrosive solutions containing hexane (10% *v*/*v*), diethyl ether (10% *v*/*v*), chloroform (CHCl_3_), dimethyl sulfoxide (10% *v*/*v*), potassium chloride (0.1 M, 0.74 wt%), and calcium chloride (0.1 M, 1.1 wt%) for 24 h. The pH solutions (pH 3, 5, 7, 9, and 11) were applied for 36 h.

### 2.7. Oil/Water Separation Tests

The oil/water separation performance was tested using a homemade apparatus The prepared membrane (52.97 mm length × 43.66 mm width × 0.89 mm thickness) was fixed between two cylindrical tubes of 40 mm in diameter. Before the separation process, the membrane was wetted with distilled water. An oil (dichloromethane, chloroform) and water (1:1, *v*/*v*) mixture was poured onto the LF-RS membrane for the separation test, with Sudan III staining the oil red and methylene blue staining the water blue. The efficiency of oil/water separation (% η) and the flux through the membrane (*Flux*, L m^−2^ h^−1^) were determined using Equations (1) and (2) as follows:

(1) $$\eta (\%)=\frac{m_{s}}{m_{0}}\times 100$$

(2) $$\begin{array}{l} Flux(Lm^{-2}h^{-1})=\frac{V}{St} \end{array}$$

where *m*_0_ stands for initial oil volume and *m_s_* stands for final oil volume. *V* stands for the volume of the filtrate that has permeated (in *L*), *S* represents the effective contact area of the membrane (in m^2^), and *t* denotes the time taken for the separation process (in hours).

### 2.8. Simulation

The cellulose Iβ model, chosen for its structural relevance to biomass materials, was created based on lattice structure data obtained from synchrotron X-ray and neutron diffraction [43]. The selected cellulose cluster model was extracted from the (110) crystal plane of cellulose Iβ, which captures critical surface interactions significant for studying hydrophobic modifications.

The dispersion of water and oil on the surfaces of the modified and unmodified cellulose membranes was analyzed through molecular dynamics (MD) simulations using Materials Studio software(V5.5 biovia, San Diego, CA, USA). Interaction energy, including van der Waals and electrostatic interactions between the cellulose membranes, was simulated using a COMPASS force field to study the effects of H^+^ and OH^−^ (H_2_O) content on surface stability. The methodology for selecting the cellulose Iβ model was based on its known structural properties, which make it representative of native cellulose. The molecular dynamics simulations were performed using the Forcite module. The model was geometrically optimized and annealed five times before executing the dynamics simulations at 500 ps under the NVT system. This allowed for a comprehensive analysis of the membrane stability and interaction dynamics.

## 3. Results and Discussion

### 3.1. Characterization of Prepared LF-RS Membrane

The structural characteristics of the prepared membranes are summarized in Table 1. After modifying the O-LF-RS membrane, the median pore diameter was 181.87 nm, with a porosity of 51.40%, higher than that of O-LF-RS alone (49.03%).

**Figure 2:**
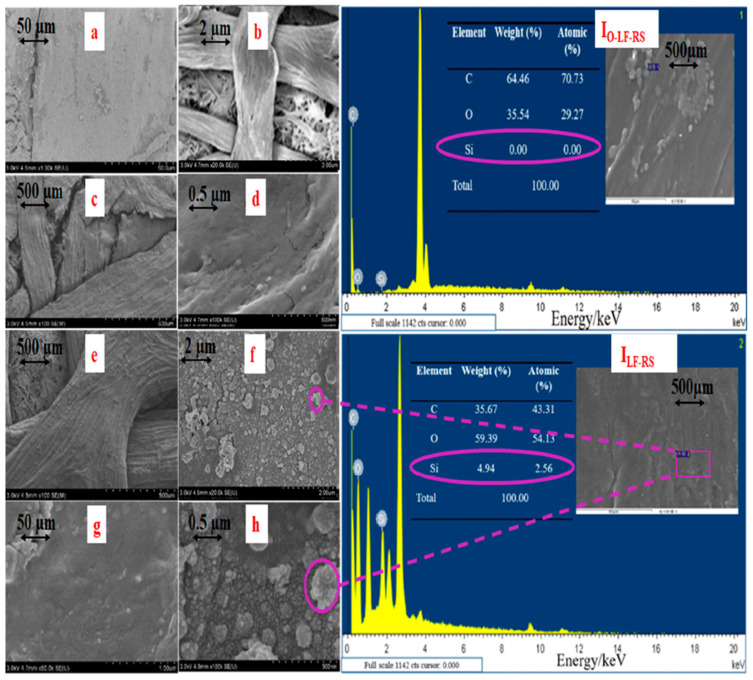
The microscopic structure and EDS-SEM analysis of the original O-LF-RS and LF-RS composite membrane: (**a**–**d**) the original O-LF-RS and (**e**–**h**) the LF-RS composite membrane. I_O-LF-RS_: EDS spectrum of O-LF-RS; I_LF-RS:_ EDS spectrum of LF-RS. (Note: The purple circles in Figure 2f,h show the formation of nanoparticles on the surface of LF-RS, and the percentage of silicone on the surface is indicated in the table circled in purple).

Surface examination was performed using Energy-Dispersive Spectroscopy (EDS) in conjunction with Scanning Electron Microscopy (SEM), as illustrated in Figure 2a–h. In Figure 2a–d, the original unmodified O-LF-RS membrane exhibited a smooth surface with a direct porous internal structure. Notable wrinkling was observed on its surface (Figure 2c,d), likely due to the overlay and hot pressing of PDM on DOR (as described in Section 2.2 and depicted in Figure 1). Conversely, Figure 2e shows that the LF-RS membrane features a rough texture on its fiber.

Figure 2f–h demonstrate the formation of numerous nanoparticles on the surface of LF-RS at magnifications of 2 µm, 50 µm, and 0.50 µm, respectively. This phenomenon can be attributed to the hydrolytic condensation of dodecyltriethoxysilane (DTES) and tetraethylorthosilicate (TEOS) with the hydroxyl groups of cellulose. The alkoxysilane groups in TEOS and DTES react with ethanol to form silanol species (Si-OH), which undergo condensation to create Si-O-Si bonds. This surface modification is likely to have increased the cross-linking density, resulting in a smoother texture and the formation of microspheres that aggregate into micro- and nanocomposite structures, enhancing surface wettability.

Figure 2 (I_O-LF-RS_, I_LF-RS_) and the EDS-SEM analysis for both types of membranes confirm the presence of Si, O, and C elements. The O-LF-RS membrane showed no Si (0%) and had a proportion of O at 64.46%. In contrast, the LF-RS membrane contained higher proportions of Si (4.94%) and O (59.39%), thus confirming the successful silicone coating on the LF-RS membrane.

**Figure 3:**
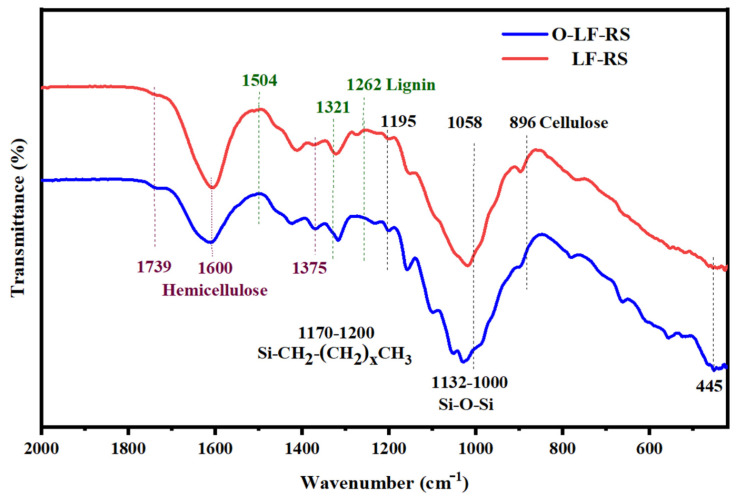
The FTIR spectra of O-LF-RS and the LF-RS membrane. (Note: The peaks related to hemicellulose are labeled in purple (1739, 1600, and 1375 cm^−1^), while those related to lignin are labeled in green (1504, 1321, and 1262 cm^−1^)).

The Fourier Transform Infrared (FTIR) spectra of O-LF-RS and LF-RS samples are shown in Figure 3 and detailed in Table 2. Distinct peaks at 1739, 1375, and 1243 cm^−1^ indicate the presence of hemicellulose groups, while peaks at 1504 and 1262 cm^−1^ signify lignin absorption. Typical cellulose absorptions are evident at 1321, 1058, and 896 cm^−1^ [44]. These peak variations (referent 1321, 1058, and 896 cm^−1^) highlight the preparation of a cellulose-based membrane achieved through selective processing to eliminate lignin and some hemicellulose. The Si-O-Si symmetric stretching vibrations in the 1132–1000 cm^−1^ range are accentuated and superimposed on Si-O-C bonds at 1000 cm^−1^ in the modified membrane [45]. Variations in the Si-CH_2_(CH_2_)_x_CH_3_ peak (1170–1200 cm^−1^), in conjunction with EDS spectra analysis, confirm the presence of silicon- or carbon-bonded organosilicon compounds on the membrane.

### 3.2. Surface Wettability and Chemical Stability

The surface wettability of the O-LF-RS and LF-RS membranes was measured via contact angle tests to investigate their hydrophobic properties towards water further. The LF-RS composite membrane exhibited high hydrophobicity, with a water contact angle (WCA) of 162.39°. Due to the oleophilic nature of the LF-RS membrane, oil was easily attracted to its surface. The WCA on the original O-LF-RS membrane was approximately 0°, while the superhydrophobic LF-RS membrane exhibited a WCA of 162.39°. This suggests that the LF-RS membrane exhibited both hydrophobic and oleophilic properties. The non-wetting properties and nano- and micro-structure layers of the superhydrophobic membrane promoted the creation of a solid/air/liquid interface via trapped air pockets, ensuring the immediate flow of water droplets. These features were crucial for the effective separation of water and oil [47].

**Figure 4:**
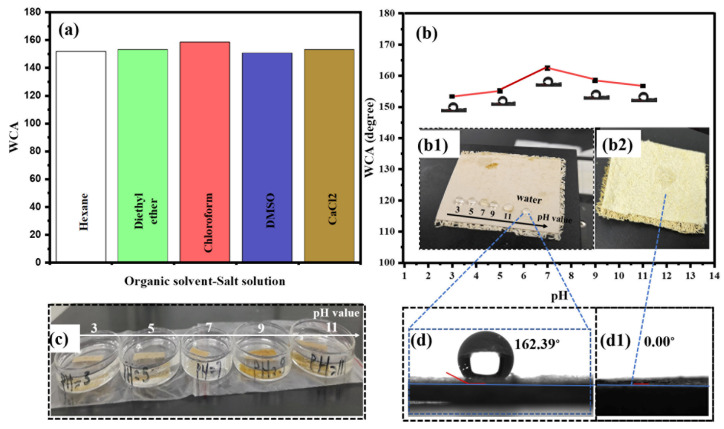
Wettability and chemical stability test: (**a**) LFS-RS water contact angle after 24 h immersion in organic and corrosive solutions; (**b**) the WCA of the LF-RS after 36 h immersion in various pH solutions; (**b1**) droplets of different pH solutions and water after 6 h; (**b2**) water droplet on the O-LF-RS; (**c**) the impregnation of the LF-RS superhydrophobic membrane in different pH solutions after 36 h; (**d**) the WCA of LF-RS 162.39°; (**d1**) the WCA of O-LF-RS.

To further verify the wettability and chemical stability of the LF-RS membrane, a series of experiments were conducted. Initially, it was immersed for 24 h in a corrosive solution containing n-hexane, diethyl ether, CHCl_3_, dimethyl sulfoxide, potassium chloride, and calcium chloride, where it maintained a water contact angle (WCA) above 150°, as shown in Figure 4a. Subsequently, the membrane was exposed to various pH solutions (3, 5, 7, 9, and 11) for 36 h, after which it retained its superhydrophobic properties, as demonstrated by Figure 4b,c. Furthermore, when exposed to acidic and basic solutions with water deposited on its surface for six hours, the membrane maintained its hydrophobic character, as indicated in Figure 4(b1),d. This remarkable chemical resistance makes it suitable for use as a filter membrane in a wide range of environmental conditions.

### 3.3. Abrasion Test

**Figure 5:**
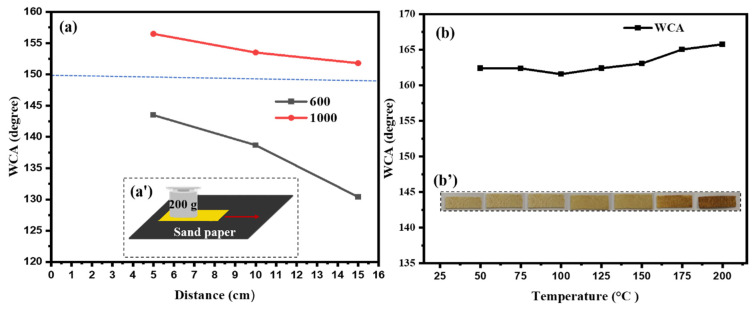
(**a**) The WCA as a function of sand paper movement; (**a’**) a schematic illustration of the methodology of the abrasion test; (**b**) LF-RS WCA as a function of heating temperature; (**b’**) the texture of LS-RS.

An abrasion test using sandpaper was conducted to evaluate the mechanical durability of the LF-RS membrane. The LF-RS membrane was placed on sandpaper sheets of different grits (600 and 1000) with a weight of 200 g on top. The surface durability was then assessed by moving it across distances of 5, 10, and 15 cm (Figure 5a). When abraded with 1000 grit sandpaper, the WCA remained at 150° for all tested distances, indicating that the coating withstood abrasive wear without significantly altering the hydrophobicity of the surface. However, with 600 grit sandpaper, the WCA stayed above 140° for 5 and 10 cm but dropped to 130.4° after moving 15 cm (Figure 5a’). This decline in WCA with coarser sandpaper grit likely resulted from increased roughness, causing more damage to the coating, which could reduce the hydrophobicity of the surface.

### 3.4. Impact of Temperature on the Contact Angle and the Texture of LF-RS

The impact of temperature is of great importance and could directly influence the efficiency of the superhydrophobic membrane in separating water from oil. To investigate the effects of temperature variations, the superhydrophobic membrane LF-RS was exposed to heat over a temperature range from 25 to 200 °C. Initially, the water contact angle remained relatively stable between 25 °C and 100 °C, maintaining a value of over 160°. However, increasing the substrate temperature to around 175 °C under heating conditions, the augmented surface diffusion favored the smoothing of surface features, leading to an increase in the WCA instead of a decrease (Figure 5b). This suggests that temperature exposure could improve surface coating stability under heating conditions. The optimized design promoted the recovery of hydrophobic properties.

The impact of temperature on the texture of the composite membrane (LF-RS) was also investigated after determining the WCAs, as previously described. Distinct shades were observed (Figure 5b’). In general, it was noted that the hue of the LF-RS composite membrane progressively changed from pale gold to dark brown with rising temperature, although the rate of change varied. For instance, between 0 and 100 °C, the color of the LF-RS membrane reacted most slowly to heat, changing from pale gold to dark gold. Then, from 100 to 200 °C, it progressively turned dark brown. At 250 °C, the LF-RS membranes showed some level of crimping, becoming brittle with scorch marks at some points, leading to the exclusion of the 250 °C sample LF-RS from subsequent experiments. The latest experiments focused instead on the properties of the LF-RS membrane at 150 °C and 200 °C.

### 3.5. TGA Analysis

**Figure 6:**
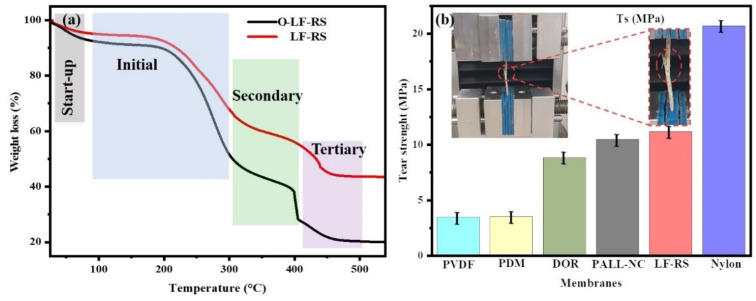
(**a**) Thermogravimetric analysis and (**b**) mechanical property.

Thermogravimetric analysis (TGA) can be used to identify phase transitions and weight losses, providing information on the thermal stability of samples under varying atmospheres. It can be seen that the two samples exhibited different thermal stability behaviors (Figure 6a). This decomposition behavior could be divided into four stages. The start-up phase occurred between 0 and 75 °C, when the original composite membrane (O-LF-RS) lost moisture earlier than the superhydrophobic membrane (LF-RS). The initial phase of decomposition occurred within the temperature range of 75–220 °C, during which the primary mass reduction stemmed from the evaporation and separation of moisture present within the material. Subsequently, a secondary decomposition stage was evident between 220 and 350 °C, marked by the breakdown of -OH groups present on the surface of the LF-RS composite material. Finally, a tertiary decomposition phase was observed beyond 350 °C, primarily associated with the combustion of the fibers themselves.

Furthermore, the composite membrane LF-RS demonstrated more excellent thermal resistance than the O-LF-RS membrane, with a difference of 22% *w*/*w*. These results indicate that LF-RS had improved thermal stability compared to the O-LF-RS membrane. This implies that the surface-grafted silicone coating underwent carbonization and persisted on the surface of the LF-RS membrane, thereby enhancing its thermal stability. The formation of a silicone coating on the surface of the LF-RS membrane was achieved using the sol–gel salinization method.

### 3.6. Mechanical Property

Tear strength tests were conducted to assess the mechanical properties of the superhydrophobic composite membrane (LF-RS), as well as the PDM and DOR. These samples were compared to three types of commercial membranes (nitrocellulose PALL-NC ref. 0.22u, PVDF membrane ref. 0.45u ISEQ00010, and Nylon ref. 0.22u RPN303B), as shown in Figure 6b. Tensile test results indicated that LF-RS has a tensile strength of 11.19 MPa, i.e., 322.47% higher than that of the PVDF membrane (3.47 MPa) and 126.58% higher than that of the nitrocellulose membrane (PALL-NC 8.84 MPa). Additionally, it is particularly important to note that the PDM (3.52 MPa) exhibited a mechanical performance similar to that of the PVDF membrane tested. The enhanced mechanical strength of LF-RS could be attributed to the three-dimensional interconnected and oriented porous structure of the loofah [48], which was further reinforced by the lamination process and the introduction of rice straw. Notably, the LF-RS membrane was less resistant than the nylon membrane (20.71 MPa).

### 3.7. The Influence of Hydrophobic Siloxane Modification on the Interfacial Distribution of Water and/or Oil (Dichloromethane) Molecules at the Membrane Surface Based on MS Modeling

**Figure 7:**
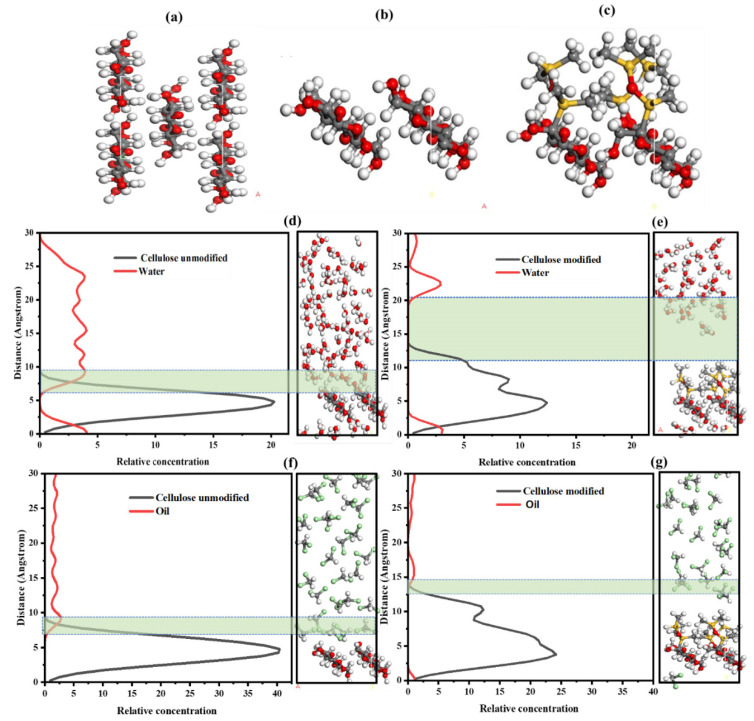
(**a**) cellulose Iβ initial; (**b**) the surface (110) of cellulose Iβ; (**c**) the modified model of cellulose Iβ; (**d**) unmodified cellulose model in water; (**e**) modified cellulose model in water; (**f**) unmodified cellulose model in oil; (**g**) modified cellulose model in oil. The green areas indicate the interfacial regions where interactions between water or oil molecules and the cellulose surface (modified or unmodified) are analyzed. These areas highlight differences in interfacial distribution and density, showing the impact of hydrophobic modification on the membrane’s behavior towards water and oil. (note: white, gray, red, and yellow balls correspond to hydrogen, carbon, oxygen, and silicon atoms, respectively).

The impact of hydrophobic modification by siloxanes on the interfacial water molecule at the membrane surface was studied. This modification led to a 3D structure on the cellulose, forming alternating hydrophobic and oleophilic layers. The cellulose-O-Si(CH_3_)_2_-O-Si(CH_3_)_2_-OSi(CH_3_)_3_ structure was formed by grafting single-chain siloxanes onto the cellulose surface after optimizing the initial model (Figure 7a,b). This structure was considered a model of hydrophobically modified cellulose (Figure 7c). Molecular dynamics simulations (using Biova Materials StudioV5.5, Biovia, San Diego, CA, USA) were employed to analyze the interaction energies between water molecules at the interface of unmodified cellulose (Figure 7d) and hydrophobically modified cellulose (Figure 7e), along with the diffusion behavior and relative positioning of water molecules on these surfaces. 

The interaction energy of water with cellulose and hydrophobically modified cellulose was calculated using the last frames of the trajectory after the system reached equilibrium. Post-equilibrium calculations revealed a significant reduction in water–cellulose interaction with the hydrophobic modification (−8.66 kcal/mol versus −234.14 kcal/mol), a decrease of 96.30%. This indicates non-bonding solid energy and mutual attraction between water and hydrophobically modified cellulose, suggesting that the hydrophobic modification weakens the interaction between water and modified cellulose, thereby improving phase separation.

The hydrophobic and hydrophilic interactions among colloids are generally associated with the interactions between particles (emulsion droplets) and the solvent that encloses them, such as water. If the droplet mimics the surface properties of water, the molecules in the surrounding space resist are expelled and oppose the approach of other particles [49]. Hydrophobic surfaces (Figure 7e) do not favor water and instead prefer direct contact with one another, thus reducing unfavorable interactions with H_2_O molecules. Consequently, hydrophilic interactions (Figure 7d) lead to a repulsive force (hydration), whereas hydrophobic interactions are attractive.

Figure 7f,g illustrates the effect of hydrophobic modification on the relative interfacial position and interaction between oil molecules on the unmodified (Figure 7f) and modified (Figure 7g) membranes. The green-shaded regions represent the interfacial zones where the distribution and interaction of water or oil molecules with the cellulose surface (modified or unmodified) are analyzed. There is a high concentration of oil molecules at the interface of the unmodified membrane under hydrophilic influence. The interfacial oil molecules in the siloxane distribution layer tend to entangle close to the cellulose membrane, forming a high-density zone (Figure 7f), while under the hydrophobic and oleophilic influence of siloxane, oil molecules exhibit a low concentration at the membrane interface (Figure 7g). Compared with Figure 7f,g, the modified superhydrophobic membrane exhibits minimal wetting behavior towards oil. Due to its superhydrophobic properties, oil does not readily adhere to the surface and instead forms compact droplets that tend to remain separated from the membrane.

In contrast, the unmodified membrane shows a more significant wetting behavior, creating a continuous layer, which can lead to more excellent oil adhesion to the membrane. This is one reason why unmodified membranes cannot separate oil while modified membranes can do so. Unmodified membranes (Figure 7d,f) in contact with water and/or oil do not repel oil and/or water effectively, allowing them to adhere more easily to the surface.

### 3.8. Oil/Water Separation Test Experiments

**Figure 8:**
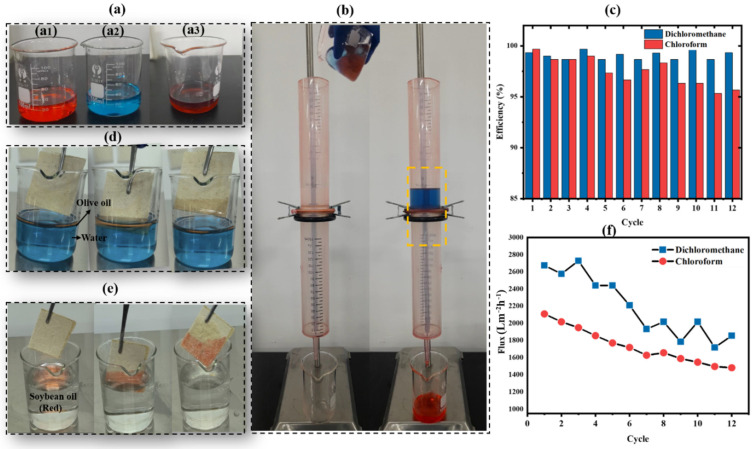
Oil/water separation test: (**a**) different solutions: (**a_1_**) red-dyed oil (dichloromethane); (**a_2_**) blue-dyed water; (**a_3_**) oil/water mixture; (**b**) separation of oil/water with LF-RS via filtration; (**c**) separation efficiency; (**d**) separation of olive oil/water by absorption; (**e**) separation of soybean oil/water by absorption; (**f**) separation debit flux.

Oil/water separation experiments were carried out with superhydrophobic membranes (LF-RS) through continuous filtration using custom-built equipment. The LF-RS membrane was placed between two gravitationally driven cylindrical tubes with an internal diameter of 40 cm. As depicted in Figure 8a–e and the Supplementary Information video (Supplementary files S1 and S2), red-colored oils (such as dichloromethane and chloroform) and blue-colored water were mixed, then poured onto the membrane, previously wetted with distilled water, until the oils were completely filtered through it. The oils were collected in a beaker below, giving an average filtration flux of 2057.37 Lm^−2^ h^−1^ for dichloromethane and 1734.69 Lm^−2^ h^−1^ for chloroform after 12 cycles. Separation efficiency was measured as 99.06% and 97.47% on average for dichloromethane and chloroform, respectively, demonstrating their high performances. As shown in Figure 8c,f, the membranes produced could be reused over 12 times for oil/water separation without any obvious separation efficiency reduction, confirming the membrane modification good stability. The LF-RS membrane showed an excellent contact angle with water, measured at 162.39°, enabling easy cleaning and restoration. Oils with densities less than that of water, such as soybean oil, olive oil, and kerosene, can be separated by adsorption after 2 cycles, with efficiencies of 7.56 (mL/g), 10.66 (mL/g), and 5 (mL/g), respectively, for the three types of oil (Table 3). The sorption performances of LF-RS membranes for these different types of oil were 5 to 10.66 times its weight, confirming its performance compared to other natural materials derived from biomass [50].

An economic analysis, along with a comparison of separation factors and water flux, was conducted for the LF-RS membrane, and three types of commercial membranes were tested, as presented in the Supplementary File (Tables S1 and S2 and Videos S1–S3).

**Figure 9:**
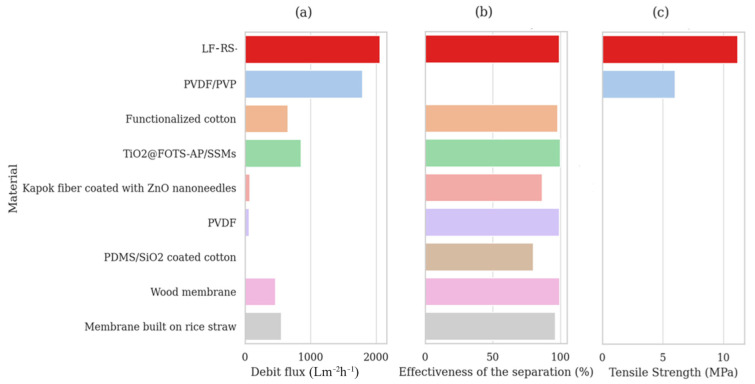
A comparison of (**a**) debit flux, (**b**) effectiveness of separation, and (**c**) tensile strength across different membranes.

Figure 9 presents an analysis limited to a few studies considering the debit flux, separation efficiency, and tensile strength. When comparing the performance of separation efficiencies and filtration flux (% *v*/*v*), the filtration rate of the current material has the highest debit flux at 2057.37 Lm^−2^h^−1^, exceeding that of PVDF/PVP (1791.4 Lm^−2^h^−1^) [51], a functionalized cotton (650 Lm^−2^h^−1^) [52], and TiO2@FOTS-AP/SSMs (850 Lm^−2^h^−1^) [53], as shown in Figure 9a. Moreover, the separation efficiency of the present material is higher at 99.06% compared to TiO2@FOTS-AP/SSMs (99.96%) [53] and PVDF (99%) [54], as demonstrated in Figure 9b. However, the wood membrane has an efficiency of 99.42% [55], while the functionalized cotton and the rice straw membrane have efficiencies of 98% [52] and >96% [56], respectively. Furthermore, in terms of strength, the RS-LF reaches 11.19 MPa, higher than PVDF/PVP (6 MPa) [51], as illustrated in Figure 9c. Thus, RS-LF shows a better filtration rate, excellent separation efficiency, and superior mechanical strength, outperforming many previous membrane studies. It is noted that the membrane exhibits potential for long-term stability even under harsh environments, attributable to the impact of membrane processing. We believe that the LF-RS membrane developed in this work performs better as an environmentally friendly material derived from natural biomass.

## 4. Conclusions

A biodegradable superhydrophobic membrane LF-RS was prepared from loofah and rice straw using an economical, fluorine-free, and environmentally friendly method. LF-RS formed by hot pressing and water activation of the top and bottom layers modified dodecyltriethoxysilane to create a superhydrophobic for oil/water separation. The as-prepared LF-RS membrane showed good chemical stability; exceptional tear resistance, being 322.47% higher than that of PVDF; and delamination resistance comparable to or even superior to tested commercial membranes such as PALL, ref. 0.22u, and PVDF membrane, ref. 0.45u ISEQ00010. Oil/water (dichloromethane) separation tests showed a remarkable separation efficiency of 99.06%, with an average filtration flux of 2057.37 Lm^−2^h^−1^ and a water contact angle of 162.39°, confirming its superhydrophobicity. In addition, the membrane could separate various types of oil (soybean oil, olive oil, and kerosene) by sorption. Molecular modeling revealed reduced interaction energies (water-membrane) by 96.30%, facilitating cleaning and restoring separation performance. This eco-friendly, cost-effective approach minimized environmental impact and secondary waste production, and its simple fabrication process suggested its feasibility for large-scale applications in industrial wastewater treatment and oil spill remediation. Future work will focus on evaluating the long-term stability of the membrane over extended cycles and optimizing the scalability of the fabrication process for industrial applications.

## Figures and Tables

**Table 1:** Membrane structure characteristics.

	Parameters	O-LF-RS Membrane	LS-RF Membrane
Intrusion Data	Median pore diameter	57.78 nm	181.87 nm
	Average pore diameter (4 V/A)	453.61 nm	2678.95 nm
Pore Structure	Permeability (mdarcy)	18,966.15	58.29
	Conductivity formation factor	0.093	0.56
	Tortuosity	3.46	60.11
	Percolation fractal dimension	2.96	2.99
	Backbone fractal dimension	2.852	2.953
Physical Properties	Interstitial porosity	47.63%	47.63%
	Porosity	49.03%	51.40%

**Table 2:** The distinctive peak observed in the FTIR spectra of the samples.

Wavenumber (cm^−1^)	Band Assignment	References
1739	Carboxyl and acetyl groups C=O stretching vibrations in hemicelluloses	
1600–1504	C=C stretching vibration in lignin	[44]
1375	C-H deformation in hemicelluloses	
1321	Vibrating and swaying CH_2_ cellulose	
1262	C-O stretch vibration in lignin	
1058	C-O stretching mainly from C(3)-O(3) H in cellulose I	
896	Vibration of C-H deformation in cellulose	
1018	Si-O-C	[45,46]
1000–1300	Si-O-Si	
1170–1200	Si-CH_2_(CH_2_)_x_CH_3_	

**Table 3:** The absorption capacity (C) of LF-RS as a function of different oil types.

Types Oil	Density ρ (g/mL)	Viscosity (20 °C, mPa S)	Sorption Capacity C (g/g)	Volume of Adsorbed Oil (mL/g) V = (C/ρ)
Olive	0.91	102	9.6	10.66
Soybean	0.92	50	7	7.56
Kerosene	0.8	2.9	4	5

## Data Availability

The authors confirm that the data supporting the findings of this study are available within the article and its Supplementary Materials.

## Supplementary Materials

The following supporting information can be downloaded at: https://www.mdpi.com/article/10.3390/membranes14110243/s1, File SA: Supplementary files S1 and S2; Table S1: Comparative analysis of flux and separation efficiency for water and dichloromethane separation using different membrane types. Table S2: Breakdown of manufacturing costs for the loofah-based membrane in CNY per square meter. File SB: Videos S1–S3: The separation of oil and water with the three types of commercial membranes was tested under the same operating conditions as the LF-RS.
