# Supplementary material for: Efficient Separation of Oil/Water by a Biodegradable and Superhydrophobic Composite Based on Loofah and Rice Straw
Source: Membranes (Basel). 2024 Nov 18;14(11):243. doi: 10.3390/membranes14110243 (PMC11596540; doi:10.3390/membranes14110243)
Supplement: Supplementary file 1 [file membranes-14-00243-s001.zip › File SA/Supplementary file Tables.pdf]

Efficient separation of oil/water by a biodegradable and superhydrophobic composite based on loofah and rice straw

Mamadou Souare[1], Changqing Dong[1,2*], Tong Xing [1,4], Junjiao Zhang[3], Xiaoying Hu[1]

[1] National Engineering Laboratory for Biomass Power Generation Equipment, School of New Energy, North China Electric Power University, Beijing 102206, China

[2] State Key Laboratory of Alternate Electrical Power System with Renewable Energy Sources, North China Electric Power University, Beijing 102206, China

[3] School of Energy, Power and Mechanical Engineering, North China Electric Power University, Beijing 102206, China

[4] Datang Environment Industry Group Co., Ltd. Beijing 100080, China

Supplementary file Tables

Table S1: Comparative analysis of flux and separation efficiency for water and dichloromethane separation using different membrane types.

Membrane	Flux ($L \cdot m^{-2} \cdot h^{-1}$)	Separation Efficiency (%)
This work	2057.37	99.06
PVDF	163.19	88.33
Nylon	122.46	78.33
Nitrocellulose NC	N/A	*Unable to filter

* The NC membrane was unable to perform the separation, making it unsuitable for this type of application.

Table S2: Breakdown of manufacturing costs for the loofah-based membrane in CNY per square meter.

	Material	Cost (CNY/m^2)
This work	Loofah	27.43
	Rice straw	3
	NaClO	17.86
	Electricity	4.8
	Water	Ignored
	Total	53.09
Commercial Membranes	PVDF	2222.22
	Nylon	2666.67
	Nitrocellulose NC	1666.67
